# Personality Genomics

**DOI:** 10.1146/annurev-psych-013125-042402

**Published:** 2025-08-11

**Authors:** Ted Schwaba, Michel G. Nivard, Elliot M. Tucker-Drob, Dorret I. Boomsma

**Affiliations:** 1 Department of Psychology, Michigan State University, East Lansing, Michigan, USA; 2 Population Health Sciences, Bristol Medical School, University of Bristol, Bristol, United Kingdom; 3 Medical Research Council Integrative Epidemiology Unit, University of Bristol, Bristol, United Kingdom; 4 Department of Psychology, University of Texas at Austin, Austin, Texas, USA; 5 Amsterdam Reproduction & Development Research Institute, Amsterdam University Medical Center, Amsterdam, The Netherlands; 6 Complex Trait Genetics, Center for Neurogenomics and Cognitive Research, Vrije Universiteit Amsterdam, Amsterdam, The Netherlands

**Keywords:** personality traits, Big Five, genetics, genomics, GWAS

## Abstract

Recent research advances have precipitated the era of personality genomics: the study of how variation in human DNA sequence predicts individual differences in characteristic patterns of thinking, feeling, and behaving. Here, we introduce personality and genomics, and we review key findings from recent genome-wide association studies of personality traits. These findings support five key observations: (*a*) sizable genetic effects on personality arise from a vast number of genetic variants with individually miniscule effects; (*b*) genetic variants associated with personality have widespread associations with other attributes, including social, economic, and medical outcomes; (*c*) genetic effects on personality generalize across groupings of people; (*d*) genetic effects on personality are minimally confounded by familial environmental effects; and (*e*) many recent genomic findings were anticipated by classic twin genetic research. For personality psychologists, embracing genomics provides unique and powerful inferential tools. For genomics researchers, incorporating unifying personality frameworks enables an integrative understanding of core behavioral dimensions.

## INTRODUCTION

Over the course of the twentieth century and into the early twenty-first, personality traits, which index relatively stable patterns of thinking, feeling, and behaving that vary across people (Roberts & Yoon 2022), were prominently featured in behavioral genetic research that compared twin pairs, adoptees, and other relatives. Such twin and family research decomposes trait variation into genetic and environmental components to study personality’s heritability, covariation, stability, and change (Bouchard 1994, Briley & Tucker-Drob 2014, Loehlin 1992, Plomin et al. 1977, Polderman et al. 2015). In these studies, the individual elements of genetic material are not measured; their associations with personality are instead inferred from the laws of inheritance and the known genetic and social relations of the study participants (Fisher 1918, Neale & Cardon 2013). Genomic methods, which aim to explain and predict human traits from the approximately three billion base pairs [adenine (A), cytosine (C), thymine (T), and guanine (G)] of our DNA sequence, had been only tentatively applied to study variation in human personality (de Moor et al. 2012, Terracciano et al. 2010). (For more information on genetic variation in humans, readers are referred to the sidebar titled Genetic Variation.) These early genome-wide association studies (GWASs) for personality traits, conducted in the early 2010s, were severely underpowered, so much so that in the last article on personality genetics published in the *Annual Review of Psychology*, Turkheimer et al. (2014, p. 534) wrote that “although GWASs have produced some notable successes in medicine…in personality it is difficult to point to any successes at all from GWASs.”

**Figure 1:**
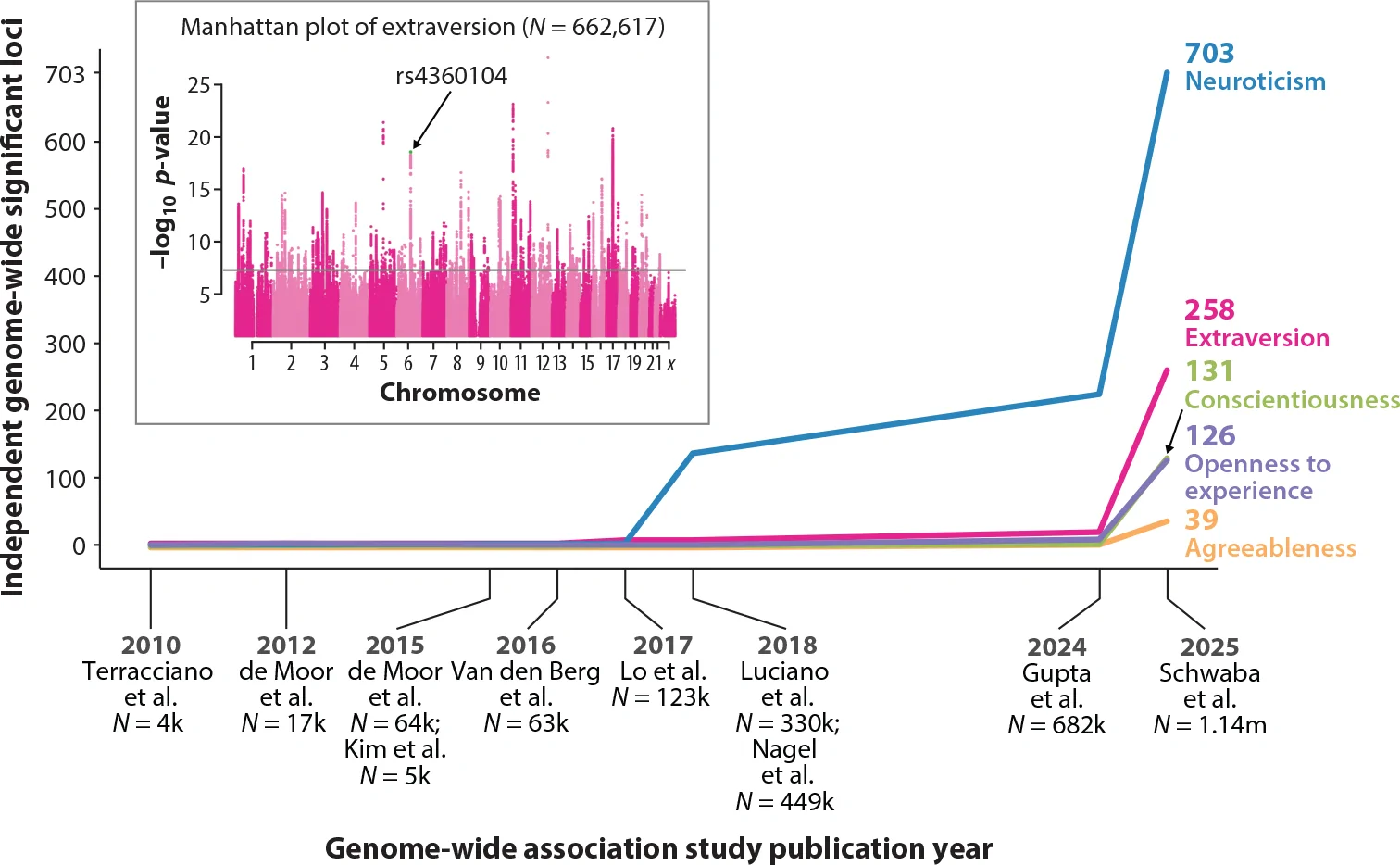
Genome-wide significant genetic associations with the Big Five personality traits over time. The line graph depicts the number of independent (i.e., approximately uncorrelated) locations on the genome that contain a genetic variant that has a genome-wide significant association (*p* < 5 × 10^−8^) with each of the Big Five personality traits over time. The labels on the x-axis denote studies conducted in that year, with the maximum sample size in thousands (k) or millions (m). The 2025 study is by Schwaba et al. (2025a). The inset Manhattan plot depicts results from a genome-wide association study of extraversion conducted by the Revived Genomics of Personality Consortium (Schwaba et al. 2025a). In that plot, the x-axis represents the position of the variant on the genome, and the y-axis represents the negative logarithm of the *p*-value of that variant’s association with extraversion (such that higher numbers are more significant). Points above the horizontal gray line are genome-wide significant at *p* < 5 × 10^−8^. One genome-wide significant variant, rs4360104, is highlighted.

In the most recent decade, major advances in personality genomics have produced an entirely different research landscape. The costs of large-scale bio-sample collections and of genotyping have dropped dramatically, allowing sample sizes to increase by orders of magnitude (Abdellaoui et al. 2023). Moreover, new statistical methods have been developed for performing genomic analysis and interrogating results (Bulik-Sullivan et al. 2015, Grotzinger et al. 2019, Mbatchou et al. 2021). These advances have enabled a recent wave of highly successful personality GWASs. We illustrate this in Figure 1, which depicts temporal patterns in the number of independent locations (loci) on the genome where a genetic variant has been associated with one of the Big Five personality traits at a significance level of *p* < 5 × 10^−8^ (0.00000005), a stringent threshold that results from adjustment for the very large number of statistical tests conducted in GWAS. A recent explosion in the number of discovered loci is apparent, driven in large part by a multigroup consortium that combined data across up to 1.14 million genotyped participants from 46 cohorts (Schwaba et al. 2025a). As we describe in this review, the success of these recent efforts is not marked simply by a boom in the number of genetic variants associated with personality (although this pattern does provide a convenient quantitative index of GWAS progress). Rather, recent genomic studies have leveraged a wide variety of methods that integrate information from many common variants across the genome to study myriad aspects of the genetic and environmental causes of individual differences in personality and their correlates (Gage et al. 2016, Pasaniuc & Price 2017). These analyses enable insights into the relevance of personality traits for reproductive and health behavior, residential mobility, family environment, mental health, and other core human phenomena. Together, these developments mark the beginning of a new era in which research on personality genetics is centered around genomic methods, and the disciplines of personality psychology and human genomics are meaningfully integrated with one another.

In this review, we cover three topics. First, we review an emerging consensus model of personality’s genetic architecture—the structured patterns of association between genetic variants and personality traits—and we consider the confounds that must be accounted for to arrive at causal estimates of genetic effects. Then, we describe how genomic approaches facilitate personality knowledge, complementing twin and family approaches by providing a unique set of tools for studying personality’s correlates, consequences, and structure. Finally, we describe how structural models of personality facilitate genomics knowledge. Habitual individual differences in thinking, feeling, and behaving influence many human attributes, and so genomic models that incorporate personality can organize and explain the pervasive patterns of genetic overlap seen among important outcomes. For the definition of several key terms used in this review, we refer readers to Table 1.

## A PERSONALITY PRIMER

Human personalities have many components, including traits, life narratives, identities, and goals (McAdams & Olson 2010). Here, we focus specifically on personality traits, like curiosity, fearfulness, and organization. Personality traits are typically measured with self-report questionnaires in which a person rates the extent to which a variety of trait features apply to them. Other methods for measuring personality, such as observing language use in the moment or asking a person’s friends and family to rate that person, tend to produce similar results as self-report questionnaires (Sun & Vazire 2019). Human languages contain thousands of personality terms (Allport & Odbert 1936), which presented a major challenge for early personality researchers; it would have been difficult for the field to make cumulative scientific progress if each scientist had studied a different set of personality traits. Conveniently, ratings on many different personality descriptive terms correlate. Individuals who are anxious tend also to be depressed and fearful, and those who tend to be organized also tend to be hardworking. Based on these patterns of covariation, an enormous effort was undertaken in the mid-twentieth century to condense and organize these thousands of traits into a systematic taxonomy (Costa & McCrae 1992a, Goldberg 1990). This resulted in the Big Five model, which has now served as the integrative, paradigmatic personality taxonomy for over a quarter century (John et al. 2008, Roberts & Yoon 2022, Soto & John 2017).

The Big Five traits are extraversion, agreeableness, conscientiousness, neuroticism, and openness to experience (most personality psychologists prefer describing the Big Five in this E-A-C-N-O order, although others prefer the acronym OCEAN). Extraversion indexes a person’s tendencies to be sociable, to be assertive, and to experience positive emotions, among other related traits; as these tend to cluster together in humans, one can study them parsimoniously in terms of a single broad factor. Agreeableness indexes traits including compassion, respectfulness, and trust. Conscientiousness includes organization, productivity, and responsibility. Neuroticism includes anxiety, depression, and emotional volatility. Finally, openness to experience includes intellectual curiosity, aesthetic sensitivity, and imagination (Soto & John 2017).Whereas each of the Big Five is typically labeled and described with respect to high scorers (e.g., extraversion describes friendly, loud people) it is important to keep in mind that each trait exists along a continuum characterized by two poles; people with low extraversion scores are introverted. Furthermore, the Big Five are part of a more general hierarchical model, with each domain composed of many more specific facets (the constituent traits described above) that are themselves composed of even narrower, contextualized trait nuances, such as one’s tendency to enjoy poetry or to leave their room messy (Mõttus et al. 2017). This hierarchical structure allows theory and research to incorporate additional specificity when necessary.

Although there are other structural models of personality used in modern research (Lee & Ashton 2004),the widespread adoption of the Big Five has proved massively useful for personality psychology (John et al. 2008). However, the Big Five are not the ground truth, nor are they universal: They are merely a useful descriptive model (Srivastava 2020). Across different languages and cultural contexts, different personality trait structures emerge, which often deviate substantially from the Big Five (Thalmayer et al. 2022). Furthermore, the Big Five are not exhaustive, as some personality traits fall outside of the Big Five (e.g., humor; Paunonen & Jackson 2000), nor are they mutually exclusive, as some narrower traits represent a combination of the Big Five (e.g., anger is a combination of low agreeableness and high neuroticism; Bainbridge et al. 2022, Costa & McCrae 1992b). These complications illustrate the inherent complexity of studying the numerous, partially overlapping traits that make up our personalities. As we discuss in this review, genomic research can provide useful further evidence about the validity and utility of the Big Five and other structural models of personality.

We have described how researchers tend to study personality traits, but it is also important to describe why. Personality traits are a core part of the human condition; there is intrinsic value in understanding individual differences among ourselves and how we came to be who we are. Why are some people kind and others aggressive, some people shy and others the life of the party? As a species, we find these differences quite salient, or we would not have created thousands of words for them. Personality traits also have explanatory utility. They shape our life paths, predicting health, wealth, work, and love lives (more on this below) as strongly as any other individual differences we can measure (Borghans et al. 2016, Roberts et al. 2007, Wright & Jackson 2023). In short, we study personality traits because they matter.

## A SHORT HISTORY OF PERSONALITY GENETICS

Over a century of research has estimated the relative contributions of genetic and environmental factors to individual differences in personality (Bakwin 1930, Eaves & Eysenck 1975, Eysenck & Prell 1951, Gottesman 1963, Pearson 1904). Historically, these investigations have relied on twin and family designs, which decompose population variation into genetic and environmental components. Genetic sources of variability are implied if pairs of individuals who tend to share more genetic material [e.g., identical or monozygotic (MZ) twins, who share 100% of their DNA, compared to fraternal, dizygotic (DZ) twins, who share on average 50% of their segregating genes] also tend to be more similar in their personality traits, keeping their shared rearing environment constant (Bouchard 1994). Environmental sources of variability can be shared or nonshared among people living together. Shared environmental effects are implied when similarity between individuals who are raised together is not fully accounted for by genetic relatedness—for example, when genetically unrelated individuals raised together in the same adoptive home resemble one another (Bouchard & McGue 1990, Tellegen et al. 1988). This shared environment includes all causes of similarity among individuals reared together beyond their genetic relatedness, including prenatal influences and the shared broad macroenvironmental exposome. Remaining sources of variation in a trait that are not attributable to genetic or shared environmental factors are termed the nonshared environment; this includes all environmental exposures that introduce differences between family members as well as measurement error and random, stochastic influences (Molenaar et al. 1993, Plomin & Daniels 1987).

A mountain of research from twin and family studies provides robust evidence that genetic variation typically accounts for approximately 30–60% of the variation in human personality traits (Polderman et al. 2015, Vukasović & Bratko 2015), with recent research suggesting that heritability is even greater if one measures personality traits from multiple rater perspectives to reduce measurement error (Mõttus et al. 2025). Perhaps surprisingly, shared environmental effects are typically absent; being raised in the same household does not tend to make people any more similar in personality (Bouchard & McGue 1990,Loehlin 1992,Tellegen et al. 1988,Turkheimer et al. 2014). The remaining 40–70% of variance in personality that is not explained by genetic factors is typically attributed to the nonshared environment (Plomin & Daniels 1987, Polderman et al. 2015, Vukasović & Bratko 2015).

Importantly, genetic, shared environmental, and nonshared environmental sources of variance must be interpreted in the context of one another. For one, their combined effects must add up to account for 100% of personality trait variance, and so an increase in one source must be accompanied by a corresponding proportional decrease in others. Additionally, unmodeled interactions and correlations between genetic and environmental factors impact their interpretation in standard twin and family models (Purcell 2002).For example, unmodeled interactions between genetic and shared environmental factors, such as when genetic predispositions toward sensation seeking are more strongly related to antisocial behavior among adolescents raised in more permissive homes (Mann et al. 2016), act like genetic factors. So too do unmodeled correlations between genetic and nonshared environmental factors—for example, if those who have a greater genetic propensity toward sensation seeking are led toward riskier environments, which in turn results in greater rates of accidental injury. Interactions between genetic and nonshared environmental factors act as the nonshared environment, and a correlation between genetic factors and shared environment acts as the shared environment. These considerations compel researchers to make nuanced interpretations of environmental and genetic effects.

During the late 1990s and early 2000s, it became financially and pragmatically feasible to measure small sets of DNA markers. Researchers began measuring sites of common genetic variation and reporting that they had confirmed hypothesized associations between genetic variants and personality traits (Caspi et al. 2003, Hariri et al. 2002). These original approaches typically tested a small number of candidate variants that had well-characterized biological mechanisms thought to be relevant to personality, such as variants in the serotonin transporter gene 5-HTT, which regulates the availability of the same neurotransmitter that is targeted by antianxiety medications (Lesch et al. 1996). The samples used to test these effects tended to be small, and the corrections for testing multiple genetic variants were sporadic. Nearly all of these findings did not replicate across studies, and large meta-analyses have come to confirm that early findings implicating commonly investigated candidate variants were largely false positives (Border et al. 2019, van de Weijer et al. 2022).

To move beyond these early approaches, researchers began measuring more genetic variants, in increasingly larger samples, to conduct large-scale searches referred to as GWASs (see the sidebar titled Genome-Wide Association Study Basics). Initial GWASs focused on biomedical outcomes, such as coronary artery disease (Craddock et al. 2007), and identified some of the first associations with common genetic variants that were truly robust (previous work had robustly identified very rare variants with large effects, often on monogenetic diseases). However, initial application of GWAS to personality traits lacked the sample sizes, and thus the statistical power, to provide major insight into the genomics of personality (Figure 1). In 2010, Terracciano and colleagues conducted a GWAS of the Big Five traits in 4,000 Italian participants but did not identify any genome-wide significant genetic variants (Terracciano et al. 2010). Neither did the first meta-analytic GWASs of the Big Five, which pooled tens of thousands of participants across multiple samples (De Moor et al. 2012, 2015; Kim et al. 2015, Van Den Berg et al. 2016).

Once personality GWAS sample sizes grew to over 100,000 in the late 2010s, statistical power was finally substantial enough that individual associations between common genetic variants [single nucleotide polymorphisms (SNPs); see the sidebar titled Genetic Variation] and personality traits began to clear the genome-wide significance threshold (Lo et al. 2017, Luciano et al. 2018, Nagel et al. 2018) (Figure 1), and studies began to provide useful insights into the molecular genetic architecture of personality traits. The largest contributor to this first wave of successful personality trait GWASs has been the UK Biobank (UKB), which includes data from about half a million genotyped middle-aged and older adults living in the United Kingdom. UKB participants completed a 12-item neuroticism questionnaire, which has been GWASed by multiple research groups (Grotzinger et al. 2019, Hill et al. 2020, Kim et al. 2023, Luciano et al. 2018, Nagel et al. 2018), as well as other questions about personality that have also been GWASed: risk-taking and adventurousness (Karlsson Linnér et al. 2019, 2021), sociability (Bralten et al. 2021), loneliness (Abdellaoui et al. 2019, Day et al. 2018), and well-being and happiness (Baselmans et al. 2019, Okbay et al. 2016). Recent progress has also been made in studying the Big Five by combining results across multiple cohorts, with over a million participants for neuroticism and over half a million participants for the other Big Five traits (Gupta et al. 2024, Schwaba et al. 2025a). Passing this half-a-million mark seems to have been the tipping point for personality GWASs: Statistical power at this sample size and above has afforded the identification of many genome-wide significant associations between SNPs and personality traits and permits statistically robust follow-up analyses of other major questions. Having caught up to the present, we now review what these GWASs have revealed about the genetic architecture of human personality traits.

## THE GENETIC ARCHITECTURE OF PERSONALITY

### Polygenicity

For all behavioral characteristics, including personality traits, genetic associations are spread within and across all 23 chromosomes. In other words, personality’s genetic architecture is highly polygenic. As early as 1918, Ronald Aylmer Fisher envisioned that the genetic architecture of an attribute like personality, which follows a normal distribution in the population, would be at least somewhat polygenic (Fisher 1918); however, it is hard to overstate the degree of personality’s polygenicity. Many thousands of genetic variants, many of which have not yet been significantly associated with a trait in GWAS research, may be relevant to personality trait variation (Schwaba et al. 2025a, Zhang et al. 2018). There is not one genetic variant for extraversion; rather, there are many, and the effects of each one are extremely small (see the sidebar titled Understanding Genome-Wide Association Study Results and the sidebar titled Manhattan Plots).As sample sizes continue to increase, some hypothesize that nearly every variant in the genome will eventually be found to have a non-null association with personality traits, whether through direct, causal influence (e.g., a SNP affects a biological substrate of personality) or through indirect influence by association (e.g., a SNP affects a variable that is correlated with personality) (Boyle et al. 2017).

### Single Nucleotide Polymorphism Heritability

Heritability refers to the extent to which individual differences in an attribute are associated with genetic variation within a population. When heritability is estimated via twin and family methods, it quantifies the aggregate (typically additive) effect of all segregating genetic variants on an attribute, expressed as a proportion of total attribute variance. When heritability is estimated from a GWAS, the definition changes subtly, albeit meaningfully, to represent the aggregate additive effect of measured and imputed common genetic variants on an attribute (as well as the effect of unmeasured variants that are indirectly indexed as a result of being in linkage disequilibrium with measured ones), expressed as a proportion of total attribute variance. This quantity, known as SNP heritability, ranges from about 5% to 15% for reliably measured personality traits, with a median of about 10% (Abdellaoui et al. 2019, De Moor et al. 2012, Gupta et al. 2024, Karlsson Linnér et al. 2021, Lo et al. 2017, Schwaba et al. 2025a, Van Den Berg et al. 2016). Because genetic effects can be contingent on environmental contexts, allele frequencies vary across populations, and alleles may interact with one another (more on these topics below), SNP heritability varies across samples (Schwaba et al. 2025a), which makes it both challenging and by-and-large uninformative to search for a single, true SNP heritability estimate for a given personality trait (Tropf et al. 2017). Furthermore, there is not any consistent evidence suggesting that SNP heritability varies systematically across different personality traits, matching findings from twin and family research (Vukasović & Bratko 2015).

Compared to the heritability estimates of 30–60% contributed by twin and family research, this ~10% estimate for SNP heritability is much smaller. What to make of this missing heritability (Maher 2008), which is seen not just for personality but for nearly all GWASed attributes (Yang et al. 2015)? We review three reasons that explain why this missing heritability gap may be the product of study methodology. First, the 10% SNP heritability estimate refers solely to effects of measured SNPs, not to variation in the entire genome. Some missing heritability is likely found in the effects of genetic variants that vary among only a small portion of the population (rare variants) as well as other forms of genomic variation (e.g., structural variants) that are not typically measured or well indexed by common variants (Wainschtein et al. 2022, Weiner et al. 2023, Wu et al. 2025).

Second, the genetic effects measured in a GWAS are estimated based on an additive model, thus failing to fully capture variation that arises from nonlinear and interactive effects (Palmer et al. 2023, Smith et al. 2024). It is possible that the alleles of a particular SNP have nonadditive effects (i.e., they could be recessive or dominant, such that the effect of inheriting two copies of a given allele is more than twice that of inheriting one copy). Additionally, the effect of a particular variant may be contingent on other variants (an interaction termed epistasis) or on the environmental context (i.e., gene × environment interaction). Unfortunately, statistical interaction effects tend to be difficult to estimate accurately (Gelman 2018), and testing nonadditive effects across the genome leads to millions of additional statistical tests that must be corrected for, making these investigations challenging at present (Lundberg et al. 2023; cf. Palmer et al. 2023, Smith et al. 2024). Twin and family research indicates that these effects may be lurking: These studies often find nonlinear patterns of heritability when comparing different relatives (Penke & Jokela 2016). For example, identical twins raised together tend to be more than twice as similar in their personality traits as fraternal twins raised together, a pattern that is inconsistent with a strictly additive genetic model (Boomsma et al. 2018, Tellegen et al. 1988) and rarely found for other attributes and outcomes (Polderman et al. 2015). These nonlinear patterns may alternatively be the product of social processes, such as assimilation and contrast effects in the ways that relatives respond to survey questionnaires (Briley et al. 2018, Kandler et al. 2010).

Third, estimates of heritability, including SNP heritability, are affected by the reliability of personality trait measurement. Measurement error will result in attenuated heritability estimates. Indeed, personality questionnaires that possess greater reliability also tend to show greater SNP heritability (Schwaba et al. 2025a),a pattern that is expected from psychometric theory (Spearman 1904) and is also empirically found in twin and family studies (Jang et al. 1998, Kandler et al. 2010). As large-scale biobank research incorporated into personality GWAS often relies on abbreviated or single-item trait measurement (e.g., Karlsson Linnér et al. 2021), genome-wide research can be especially vulnerable to this form of heritability attenuation. After accounting for these three methodological differences, the heritability gap between genomic methods and twin and family methods may shrink considerably, as was demonstrated for research on the heritability of human height (Yang et al. 2015, Young 2022; cf. Matthews & Turkheimer 2022).

### Pleiotropy

Another important characteristic of genetic architecture is pleiotropy, that is, the extent to which genetic variants associated with a certain attribute are also associated with other attributes (Solovieff et al. 2013). Pleiotropy may arise, for example, when the same biological pathways are involved in the development or expression of two different traits or when one trait has an (indirect or direct) effect on another trait that causes genetic effects on the former to become associated with the latter. One straightforward indication of pleiotropy is when GWASs of separate traits find significant associations with the same SNP. For example, a large-scale GWAS of externalizing behaviors, which are relatively consistent behavioral patterns related to self-regulation, identified a genome-wide significant association for rs12740789, a variant in the Neuronal Growth Regulator 1 (NEGR1) gene (Karlsson Linnér et al. 2021). In a different study, this SNP was associated with a decreased heart rate response to recovery after exercise (Verweij et al. 2018). This variant thus appears to index a pleiotropic effect. Overall, of the 579 lead SNPs that were significantly associated with externalizing behaviors in that study, 93% percent were in locations of the genome that had previously been associated with other GWASed attributes (Karlsson Linnér et al. 2021).

Pleiotropy is also indicated by genetic correlations (*r*_*g*_s), which index the shared heritability across attributes estimated using all available SNPs in the corresponding GWASs and not simply SNPs that surpass the genome-wide significance threshold. Genetic correlations can be interpreted similarly to a standard phenotypic correlation, keeping in mind that the genetic correlation indexes the associations between genetic propensities for the pairs of attributes, not the attributes themselves. Returning to externalizing behavior, genetic variants associated with externalizing were also substantially associated with conceptually similar personality traits, like high adventurousness; with pathologically relevant behaviors, like suicide attempts; and with life outcomes, like lower educational attainment in adulthood (Karlsson Linnér et al. 2021).This provides further evidence for pleiotropic effects on personality and a variety of external outcomes.

Finally, pleiotropy can be indicated using polygenic indices (PGIs) to predict novel external traits. PGIs are genetic summaries that aggregate many (often millions of) SNP associations with a trait, as estimated in a GWAS, into a single number for each individual participant in an independent data set (Burt 2024). Karlsson Linnér et al. (2021) used genome-wide associations with externalizing behavior from the UKB data set to derive PGI weights, which they then applied to participants in the National Longitudinal Study of Adolescent to Adult Health. Participants in that study who inherited a greater liability for externalizing behavior (a higher externalizing PGI) were more likely at some point in their lives to be arrested, to use illicit drugs, and to be fired from work.

These examples specific to externalizing behavior exemplify the broad patterns of pleiotropy found across all personality traits (Brainstorm Consortium et al. 2018, Gupta et al. 2024, Schwaba et al. 2025a). Genetic variants associated with personality are associated with a plethora of attributes spanning everyday behaviors, physical and mental health and illness, and consequential long-term life outcomes.

### Generality

As we discussed, the Big Five are not a universal, ground truth but merely a useful taxonomy. It may be that the genetic architecture of the Big Five varies across different groupings of people. For example, twin and family studies have indicated that the heritability of personality traits decreases with age (Briley & Tucker-Drob 2014, Kandler et al. 2021) and may vary depending on whether personality is measured using self- or peer reports (Briley & Tucker-Drob 2014, Kandler et al. 2010).

Initial tests using GWAS data have provided further insight into the generality of personality traits’ genetic architecture by using genetic correlations to estimate the similarity of SNP–trait associations across Western geographic regions, personality questionnaires, reporter perspectives, military veteran statuses, and age groups (Schwaba et al. 2025a). Correlations across these groupings were high, albeit imperfect (average *r*_g_ = 0.86). In other words, there was a strong tendency for SNPs associated with being more conscientious in one’s teens to also be associated with being more conscientious in one’s sixties and for SNPs associated with a person self-reporting high neuroticism to also be associated with close others rating that person as highly neurotic. The genetic architecture of personality traits appears to be broadly generalizable, although follow-up across a broader range of groupings is necessary to further quantify this generality.

These comparisons also identified one potential exception to this pattern of generality: Genetic associations with agreeableness varied across groups to a greater degree than associations with the other Big Five, as indexed by smaller cross-group genetic correlations (mean cross-group *r*_*g*_ = 0.72 versus 0.90 for the other Big Five; Schwaba et al. 2025a).We are intrigued by this finding, which may represent differentiated cultural mechanisms through which genetic variants produce personality trait perceptions. To illustrate this idea with a contrived example, suppose that certain genetic variants cause individual differences in sarcasm in both the United States and the United Kingdom. However, if sarcasm was culturally perceived in the United States as more disagreeable than in the United Kingdom, genetic associations with agreeableness would differ across the two countries.

### Follow-Up and Biological Characterization

One can further qualify an attribute’s genetic architecture by characterizing the biological functioning of the SNPs associated with it, leading to a more integrated biopsychosocial understanding of personality and human behavior. Of the many genetic variants associated with personality traits, a few focal SNPs have garnered attention in recent high-powered genomic research. SNPs in and around the Cell Adhesion Molecule 2 (CADM2) gene have been linked to a very broad set of impulsivity-related personality traits, including risk-taking, adventurousness, attention-deficit/hyperactivity disorder (ADHD) symptoms, and problematic alcohol and tobacco use (Pasman et al. 2022, Sanchez-Roige et al. 2023). SNPs in and around the Forkhead Box Protein P2 (FOXP2) gene have been associated with borderline personality disorder liability (Streit et al. 2024) and with each of the Big Five besides openness to experience (Schwaba et al. 2025a). Among other functions, both CADM2 and FOXP2 are involved in synaptic plasticity, especially in the frontal cortex and striatum regions of the brain. These genes may modulate a person’s learning in the context of rewards, linking genes to personality through their social experience. These are just a few notable examples of how the biological functions of individual GWAS discoveries for personality have been probed. With the recent explosion of GWAS discoveries for the Big Five (Figure 1), amounting to well over 1,000 trait-associated independent loci, one could write dozens of papers simply on the biological functions implicated by individual SNPs.

The biological mechanisms implicated by GWAS can be studied more holistically by subdividing variants with similar biological properties into different sets and then examining which gene sets exhibit disproportionate amounts of trait-associated genetic signal relative to their sizes. For example, SNPs expressed in the brain account for a disproportionately large amount of personality’s heritability, whereas SNPs expressed outside the brain, for example in the thyroid, blood cells, or skin, account for essentially none (Bryois et al. 2020, Hill et al. 2020, Karlsson Linnér et al. 2021). Finer-grained gene sets can provide more meaningful inferences while still enabling researchers to aggregate information across constellations of genetic variants. Recently, Schwaba et al. (2025a) reported that, across the Big Five, SNPs in gene sets that are intolerant to protein-truncating mutations were disproportionately relevant to personality heritability, a link that has also been reported for psychiatric disorders such as schizophrenia (Akingbuwa et al. 2022). Across brain gene sets, specific patterns of gene set enrichment were correlated highly but imperfectly with the Big Five, which points toward both common and differentiable neurobiological substrates for different personality traits (Schwaba et al. 2025a). The biology of personality has long been enigmatic (Hyatt et al. 2022, Sundin et al. 2021), but the biological characterization of genomic findings appears promising for making further progress on this topic.

## CONFOUNDING

GWAS associations represent a nondeterministic statistical likelihood that a person who has inherited certain alleles will score higher or lower on, e.g., a personality trait questionnaire. Understanding whether these associations are causal requires considering alternative explanations for their origin. A vast amount of theory and research has been devoted to identifying and quantifying potential confounds in genomic research (e.g., Madole & Harden 2023, Veller & Coop 2024) and finding methodologies to surmount them (e.g., Atkinson et al. 2021, Howe et al. 2022, Tan et al. 2024). Here we consider two potential confounds to GWAS that have received particular attention within the field at large, and we review results from recent studies of personality genomics that have leveraged innovative designs to test and correct for these confounds.

### Population Stratification

Genetic effects on a trait may be confounded by differences in allele frequency within and across groups of people. Specifically, when groups of people that share more common alleles also tend to share environmental and cultural conditions, any difference in the means of a GWASed trait between different groups will come to be spuriously associated with SNPs whose allele frequencies differ between them. This phenomenon is known as population stratification (Cardon & Palmer 2003).

Personality research outside of genetics has found that personality is correlated with culture and environment, flagging population stratification as a possible consideration in personality genomics. An individual’s personality is associated with where they live, from the macro country level (e.g., people living in the US Midwest tend to score lower on openness to experience than their coastal countrymates; Ebert et al. 2022) to the micro city level (e.g., people living in different London neighborhoods have different average levels of openness to experience; Jokela et al. 2015). Furthermore, as a result of both long-term and recent human migration histories, individuals residing more proximally to one another tend to share more recent common ancestors and thus have more similar allele frequencies (Novembre & Stephens 2008). As a result, without appropriate controls, a GWAS that samples people from varying geographies may find spurious genetic associations with personality.

Multiple steps are taken in GWAS that reduce the confounding effects of population stratification. GWAS are typically conducted separately within broad groups with similar recent genetic ancestry (e.g., within people with broadly European-like, African-like, and East Asian–like genomes). Further control variables that capture major axes of genetic variation are then added within each of these groups; these controls address more subtle dimensions of population stratification, such as latitude and longitude within Europe (Novembre & Stephens 2008). In addition, some methods for quantifying SNP heritability and genetic correlations employ estimators that are robust to uncontrolled population stratification effects (Bulik-Sullivan et al. 2015, Young et al. 2022).These steps considerably reduce the effects of population stratification, although some fine-grained stratification often remains (Abdellaoui et al. 2022).

An important caveat to the above discussion is that associations between genetic variants and the environment are not always indicative of simple confounding. Often, they may constitute a causal mechanism linking genetics to personality. For example, a person’s inherited disposition may cause them to seek out certain environments that then influence their subsequent personality trait expression (referred to in the twin and family literature as active gene–environment correlation; Plomin et al. 1977). In support of this, Schwaba et al. (2025a) found that people who inherited a genetic profile associated with higher openness to experience tended to move into cosmopolitan neighborhoods by midlife, which might facilitate further increase in their openness. People who inherited a genetic profile associated with greater neuroticism, on the other hand, tended to move into more economically challenged areas, which might in turn cause higher neuroticism (Schwaba et al. 2025a). Genes, environments, and personality traits are interrelated in circuitous, complex ways.

### Assortative Mating

People who are similar are often more likely to mate with one another. This phenomenon, known as assortative mating, leaves its mark on the genome by introducing correlations between distal genetic variants, and it can bias heritability estimates in twin and family research as well as genomic studies (Horwitz et al. 2023, Okbay et al. 2022).

For personality traits, there is remarkably little assortative mating. On average, phenotypic personality research has not found that birds of a feather flock together or that opposites attract: The personality traits of two parents are only slightly correlated (Horwitz et al. 2023). By estimating genetic correlations between relatives, one can make inferences about assortative mating that go back several generations (Sunde et al. 2024). However, Schwaba et al. (2025a) found that assortative mating is, and has been, small or undetectable for most of the Big Five. Of the Big Five, openness to experience exhibited genetic correlations between spouses, siblings, and cousins that deviated most from what one would expect given their genetic relatedness, albeit still to a relatively minor degree. This pattern was anticipated by phenotypic research spanning different cultures (McCrae et al. 2008), and it is consistent with openness’ associations with cognitive ability, political preferences, religiosity, and educational attainment, four traits for which assortative mating tends to be especially strong (Horwitz et al. 2023). Overall, assortative mating is not an important source of confounding in personality genomics.

### Within-Family Genome-Wide Association Studies

To evaluate the overall extent of uncontrolled confounding in personality genomics, researchers have combined twin and family methods with GWAS by sampling related individuals from family units and estimating genome-wide associations while controlling for the genotypes of an individual’s family members (Howe et al. 2022, Tan et al. 2024, Young et al. 2022). For example, because full siblings share on average 50% of their segregating genetic material at any given position on the genome, one sibling may inherit one effect allele and one noneffect allele, whereas another sibling may inherit two effect alleles and zero noneffect alleles (see the sidebar titled Genome-Wide Association Study Basics for more information). This sibling difference in the number of effect alleles can then be associated with an attribute. When such association tests are estimated among siblings reared together, these within-family GWAS designs eliminate confounding due to population stratification and very substantially reduce confounding associated with assortative mating. They also address another potential confound, shared rearing environment, introduced above in the section titled A Short History of Personality Genetics (Tan et al. 2024, Veller & Coop 2024). Further, the Mendelian laws of genetic segregation specify that, for a given segment of DNA, which alleles an individual inherits from the mother’s pair and which alleles that individual inherits from the father’s pair is random. Because of this, sibling genetic comparisons reflect random assignment to genotype, constituting a randomized experiment. Comparing the results of within-family GWASs with those of standard population-level GWASs among unrelated individuals, thus, fully reveals the role of confounding in genetic associations with personality.

For the Big Five personality traits, the results of within-family and population-level analyses have been highly consistent with one another. SNP heritability estimates from within-family GWASs are only slightly attenuated, or not attenuated at all, compared to population-level GWASs (Howe et al. 2022, Schwaba et al. 2025a, Tan et al. 2024). Furthermore, genetic correlations between within-family GWASs and population-level GWASs are extremely high, and they are significantly less than 1 only for neuroticism and agreeableness in research to date (Howe et al. 2022, Schwaba et al. 2025a, Tan et al. 2024). Finally, using PGIs constructed from population-level GWASs, sibling differences in PGIs predict personality traits just as strongly as PGIs predict personality traits among unrelated individuals (Schwaba et al. 2025a). These results confirm that patterns of SNP–trait associations for the Big Five are largely unbiased by confounds associated with population stratification and assortative mating as well as shared rearing environment (as expected from past twin and family research). Qualifying this pattern, similar analyses of personality traits with close relevance to psychiatric disorders, such as externalizing behavior and depressive symptoms, indicate a greater degree of confounding (Howe et al. 2022, Tan et al. 2024, Tanksley et al. 2025), which is consistent with twin and family studies that have found somewhat stronger shared environmental effects and assortative mating for these traits (Horwitz et al. 2023, Kendler et al. 2015, Polderman et al. 2015).

Overall, these recent genomic findings substantiate a minimally confounded model of the genetic architecture of personality traits. However, challenges do remain in causal inference, such as identifying which variants within a block of coinherited SNPs are causal (Hutchinson et al. 2020) and through what biological, developmental, and/or social mechanisms they act.

### The Next Decade

We summarize five key observations we have made regarding personality’s genetic architecture in Table 2. It is clear from these observations that the results of recent research in personality genomics have already paid major dividends in our understanding of heritable variation in personality traits. We expect that, in the next decade, as personality genomics expands in inquiry, we will learn much more to clarify these observations.

Several paths for future investigation appear especially promising. The first is developing inferential precision across traits, including the Big Five, narrower facets, and contextualized personality nuances. The genetic architecture of personality may shift as we consider traits with more specific content, and, as GWAS results for agreeableness suggest, individual traits may hold their own architectural quirks. Second, as methods for measuring the genome continue to improve, it will become increasingly feasible and valuable to interrogate the effects of rarer genetic variants and structural variants, which to date have been almost completely absent from personality genomics (cf. Wu et al. 2025). Research on other attributes suggests that this variation may have strong effects on personality (Wainschtein et al. 2022, Weiner et al. 2023). Third, there are many opportunities to identify and elucidate the role of confounding factors in personality genetics, such as assimilation and contrast effects among siblings (Briley et al. 2018, Kandler et al. 2010).

## THE VALUE OF GENOMICS FOR PERSONALITY PSYCHOLOGY

Just as findings from, for example, longitudinal studies of personality development have permeated and informed nearly all aspects of personality psychology throughout the last decade (e.g., Costa et al. 2019, Roberts & Yoon 2022), so, too, can results of personality genomic research facilitate a more comprehensive understanding of personality writ large. In this section, we describe how genomic methods provide unique inferential angles into personality’s correlates, consequences, and structure that other methods cannot yield.

### Studying Personality’s Correlates with Genetic Data

Personality psychology is fundamentally oriented toward correlations between individual differences as a method of inquiry. Genetic correlational estimates derived from GWAS data permit a considerable extension of this methodology. Genetic correlations often reproduce their phenotypic counterparts, a phenomenon that has been referred to as Cheverud’s conjecture (Cheverud 1984) or the phenotypic null hypothesis (Turkheimer et al. 2014). For example, Schwaba et al. (2025a) reported widespread and substantial genetic correlations between neuroticism and liability for each of 10 psychiatric disorders, consistent with phenotypic results that implicate neuroticism as being central to psychopathology (Barlow et al. 2014). However, there are also many instances in which patterns of genetic and phenotypic correlation diverge considerably. For example, nearly all of the psychiatric disorders investigated by Schwaba and colleagues were also positively genetically correlated with openness to experience, a result that contrasts strongly with the null correlations that are typically reported in phenotypic analyses (Widiger & Crego 2019). There are multiple potential explanations for this discrepancy. One is that associations are nonlinear, such that the genetic variants associated with severe psychopathology are associated with openness to experience only among asymptomatic individuals in the normative range of functioning, whereas comparisons of openness to experience between asymptomatic and symptomatic individuals (which serves as the basis for phenotypic correlations) capture a more extreme region of the bivariate distribution in which this trait is not associated with mental health. A related explanation is the presence of antagonistic pleiotropy, a process by which genetic variants increase evolutionary fitness with respect to one trait while reducing fitness for another trait. For example, genetic risk for schizophrenia is associated with increased creativity among first-degree relatives of individuals with a schizophrenia diagnosis (Kyaga et al. 2011, Power et al. 2015). Comparing patterns of phenotypic and genetic correlations thus promotes a deeper understanding of both.

In contrast to phenotypic correlations, which require that the variables being correlated be measured in the same individuals, genetic correlations can be estimated when the variables being correlated have been GWASed in different participant samples. This is possible because a genetic correlation indexes the extent and direction to which genetic variants predictive of one attribute are predictive of another attribute. This allows genetic correlations to test personality associations that are difficult or impossible to estimate with phenotypic data. For example, borderline personality disorder (BPD) is characterized by instability in mood, identity, and interpersonal relationships, and it is highly associated with neuroticism (Streit et al. 2024). Studying overlap between this disorder and others, such as posttraumatic stress disorder (PTSD),can provide clues into the etiology of personality pathology. However, because diagnosis of these disorders is relatively rare, it is extremely difficult to ascertain a participant sample for studying their phenotypic correlation (i.e., their comorbidity). In contrast, it is straightforward to estimate the genetic correlation between these two disorders using results based on two separate GWASs. Such an analysis reveals a high degree of genetic sharing between BPD and PTSD (*r*_g_ = 0.77), and between BPD and major depressive disorder (*r*_g_ = 0.74) (Streit et al. 2024), illustrating BPD’s close ties to internalizing disorders.

Genetic correlations can also provide unique information about the similarity of genetic architecture across distinct groupings of individuals. For instance, sex-stratified GWASs of psychological attributes, including neuroticism, have found a high degree of similarity across sexes, as indicated by genetic correlations between cisgender males and females at or near 1 for each trait (Bernabeu et al. 2021, Schwaba et al. 2025b). This finding generalizes to attributes whose average scores tend to differ by sex. Though women tend to score higher on neuroticism than men, genetic mechanisms that give rise to within-sex individual differences in neuroticism are largely sex-general. Importantly, no analogous cross-sex correlation can be derived in phenotypic data. This same cross-group approach has also been used to examine the stability of genetic effects on an attribute across ages, which would require a many-decade longitudinal study to be investigated phenotypically. For instance, the genetic correlation between childhood and adult cognitive ability has been estimated at *r* = 0.89 in data from separate child and adult GWASs (Sniekers et al. 2017). Meta-analyses of longitudinal twin and family studies of personality indicate that the temporal stability of genetic contributions to personality may follow different patterns from those observed phenotypically (Briley & Tucker-Drob 2014, Costa et al. 2019, Kandler et al. 2021), highlighting another key divergence between genetic and phenotypic correlations that is worth probing further with genomic methods.

Finally, genomic methods enable inferences about personality in genotyped cohorts for which phenotypic personality data are not available, as personality PGIs can be computed from genotype data alone (using weights derived from an external GWAS of personality). For example, the inferences about assortative mating and residential mobility described in the section titled Confounding (Schwaba et al. 2025a) were derived from participants of the UKB, who did not contribute phenotypic data on any Big Five trait besides neuroticism. Based on participants’ genotype information, Schwaba and colleagues were nonetheless able to construct PGIs for each of the Big Five to study questions about personality in this 500,000-person sample with rich geographic measurement. This feature further expands the scope of personality investigation to many more data sets and large-scale biobanks, which often administer brief personality measures or omit them entirely.

### Testing Causal Models of Personality

It is challenging to test causal models of personality traits using standard research designs, like randomized controlled trials (Grosz et al. 2020). This is partially because personality traits are highly stable over time and context (Costa et al. 2019),which makes them difficult to manipulate in brief experiments. It is also because the effects of personality traits on key life outcomes may arise through the consistent accumulation of small effects over many years, making short-term studies of personality change inappropriate for testing long-term causal processes (Wright & Jackson 2023). Certain properties of the genome afford novel opportunities for causal insight into long-standing research questions central to personality psychology.

One of these research question concerns the back-and-forth debate waged over the last 50-plus years into the ontological status of personality traits. Is personality’s structure created by broad causal factors, such as neuroticism, that induce covariation among narrower traits, such as anxiety, depressiveness, and emotional volatility? Or do narrower traits dynamically interact with one another—such that being depressive makes a person feel anxious, which causes them to act in emotionally volatile ways, in turn exacerbating depression—and factors like neuroticism are an emergent property of these interactions (Cramer et al. 2012, DeYoung 2015)? A method for comparing these alternative models is to first identify a set of elementary, statistically independent trait-relevant components and then test whether those components’ effects on personality act broadly across the narrower traits, as would be expected under the factor model, or rather act idiosyncratically on individual traits or subsets of traits, as would be expected under an alternative model, such as a network model (Carroll 1993, VanderWeele & Vansteelandt 2022). Genetic variants are especially well-suited for this task (Clapp Sullivan et al. 2024). They are functionally elementary, variants distant on the genome are inherited completely independently of one another, and the temporal directionality of their effects is clear.

Researchers have begun applying this method to test the validity of personality factors using genomic data (for similar approaches using twin and family data, see Briley & Tucker-Drob 2012, Franić et al. 2013). The initial application of this procedure was to neuroticism (Grotzinger et al. 2019; see also Kim et al. 2023). Many SNPs were found to have associations with each of 12 specific neuroticism items that were proportional to those items’ associations with a broader neuroticism factor, which indicates that genetic effects on personality may indeed be operating through such a factor. Furthermore, as would be expected for a hierarchical model of personality in which individual behaviors are associated with both a broad factor and specific facets, other SNP–trait associations were more narrowly acting. For example, some SNPs were associated with worry, nervousness, and tenseness but not with loneliness or guilt, indicating genetic pathways that bypass the broad neuroticism factor to have a narrower association with anxiety. Application of this approach to externalizing behavior (Karlsson Linnér et al. 2021) also indicated that many SNPs had factor-proportional associations with a variety of distinct externalizing behaviors, supporting the validity of a broad externalizing factor. Notable exceptions to this factor model were SNPs located in and near the Alcohol Dehydrogenase Gene 1B (ADH1B), which were associated specifically with problematic alcohol use but not with other externalizing behaviors. ADH1B influences alcohol metabolism, providing a concise biological explanation for this narrowly acting genetic pathway. As these techniques become more widely applied to personality traits, we are excited for their potential to inform long-standing debates about the validity of broad personality trait factors like the Big Five, on which progress has stalled for decades.

A second inferentially powerful method, known as Mendelian randomization (MR), uses genetic variants as an instrumental variable to test causal associations between two attributes (Sanderson et al. 2022). The core logic behind MR is that these genetic variants can affect an outcome only through their effects on a more proximal causal variable (e.g., genetic variants associated with nicotine metabolism are expected to affect lung cancer only via smoking behavior). Properly applied, MR can inform debates about the causal effects of behaviors, events, and experiences on personality trait development, which to date have been extremely challenging to identify (Grosz et al. 2020, Luhmann et al. 2021). MR has only tentatively been applied within personality genomics to date (Gupta et al. 2024, Rukh et al. 2023, Schwaba et al. 2025a). For MR research to result in valid causal inferences, many assumptions need to be met (Power et al. 2024),and personality traits often challenge these assumptions. For example, MR research is most straightforwardly conducted with genetic variants that have large effects on an attribute that operate through well-understood biological pathways. Common variants associated with personality traits, in contrast, have small effects and operate through complex and poorly understood pathways. Furthermore, widespread correlations between personality traits and other attributes make it difficult to infer that causal effects are due to personality traits alone and not to other potential co-occurring factors. Given the causal payoff of well-done MR research, we anticipate that future research will successfully tackle these challenges to incorporate this rapidly advancing methodology (Sanderson et al. 2022) into the personality genomics toolbox.

## THE VALUE OF PERSONALITY PSYCHOLOGY TO GENOMICS

We conclude by discussing the value that personality psychology may bring to genomics. In the last decade, an ever-increasing number of human attributes have been submitted to GWASs, producing an increasingly elaborated atlas of genetic correlations (Bulik-Sullivan et al. 2015). There are many, many patterns of genetic sharing and differentiation to make sense of. However, genomics lacks a comprehensive framework for organizing these results. In other words, our understanding of the genetic architectures underlying psychological traits, medical diagnoses, social behaviors, and major life outcomes has largely been siloed into findings of individual GWASs, with limited synthesis into cumulative knowledge (cf. Brainstorm Consortium et al. 2018). Recently, multivariate methods have been introduced into GWAS studies that enable the joint modeling of constellations of attributes based on their empirical patterns of genetic correlation (Grotzinger et al. 2019). This strategy has enabled remarkable progress in GWAS of psychiatric disorders (Grotzinger et al. 2025), undercovering transdiagnostic dimensions of genetic liability (e.g., substance use disorders, internalizing disorders, compulsive disorders) that each encompass multiple clinically distinct disorder diagnoses. For instance, the high degree of shared genetic liability to PTSD, major depression, and anxiety disorder has been modeled as a genetic factor representing cross-cutting liability to internalizing disorders; genetic variants associated with one of these disorders are often associated with all three. We propose that personality traits may provide an organizing framework for synthesizing GWAS results across an even more expansive set of traits in this way.

Many human attributes and life outcomes that can plausibly be directly or indirectly influenced by variation in a person’s patterns of thinking, feeling, or behaving have been associated with personality trait variation (Borghans et al. 2016, Roberts et al. 2007, Soto 2019). Empirically organizing these socially and behaviorally relevant GWAS phenotypes in terms of their relationships with personality traits may thus be especially informative. For example, research has found moderate genetic correlations among longevity, (lack of) drug use, and medication adherence (Cordioli et al. 2024, Deelen et al. 2019). What links these three? Each of these three factors is highly relevant to the personality domain of conscientiousness. Conscientious people are responsible, organized, and health conscious. They visit the dentist more often, check into the emergency department less frequently, and are less likely to engage in risky behaviors (Bogg & Roberts 2004, Willroth et al. 2023). Consistent differences in these conscientiousness-relevant behaviors may thus accrue over time into predictable differences in how long a person lives, their drug use, and whether they take the medications they are prescribed. One need not allude to the intricacies of molecular biology for mechanistic explanation; personality traits may, in many instances, be the most appropriate level of explanation (Smith 2011).

This strategy may also improve inferences in personality genomics, a research area where GWASs to date have often been conducted with respect to individual personality traits, siloing knowledge. For example, recent GWASs of sociability (Bralten et al. 2021) and adventurousness (Karlsson Linnér et al. 2019) situate these traits independently from Big Five extraversion, despite high genetic correlations among the three that suggest substantial shared genetic architecture. Decades of personality research have identified that sociability and adventurousness function as facets of a broader, subsuming extraversion factor, with adventurousness also displaying substantial associations with openness to experience (Costa & McCrae 1992a, Bainbridge et al. 2022). Considering these traits as they relate to a cross-cutting genetic extraversion factor affords more parsimonious knowledge generation. For example, all three have been linked to lower genetic liability for autism spectrum disorder (Bralten et al. 2021, Karlsson Linnér et al. 2019, Schwaba et al. 2025a). GWASs of personality components on the well-being spectrum, such as happiness, life satisfaction, depressiveness, and well-being, have in contrast received integrated treatments (Baselmans et al. 2019, Okbay et al. 2016, van de Weijer et al. 2022). These findings underscore that variation in nearly all personality traits can be described in terms of broad factors, like the Big Five (Bainbridge et al. 2022). Organizing personality trait genomics using broad factors can also be used to identify trait spaces for which genetic architecture has yet to be investigated. Few specific personality traits relevant to agreeableness and conscientiousness have been submitted to GWAS, despite their critical relevance to interpersonal and economic life outcomes (Bogg & Roberts 2004, Noftle & Robins 2007, Soto 2019). As personality genomics becomes increasingly established, these examples illustrate how conceptualizing traits in terms of broad factors representing cross-cutting constellations of genetic variance can productively organize the field.

## CONCLUSION

In the last decade, remarkable strides have been made in genomic research on personality traits, demonstrating the methodological and practical feasibility of this enterprise and repositioning genomics as central for inquiry into the origins of personality. We now know a great deal about how common genetic variants are associated with variation in people’s thoughts, feelings, and behaviors. These findings were anticipated by past twin and family research, have enabled recent traction on long-standing research questions, and have given rise to novel research directions not previously possible. Inferences from genomic methodologies inform our knowledge of personality’s correlates, consequences, and structure, addressing core questions relevant to all of personality psychology. Similarly, because patterns of thinking, feeling, and behaving are relevant to many human attributes, personality can serve as a useful organizing framework for many areas of genomic research. The era of personality genomics has begun.

## Figures and Tables

**Table 1:** Key terms and definitions

Term	Definition
**Unit of analysis**	
Personality	Psychological characteristics that define an individual animal in comparison to others.
Personality traits	Habitual individual differences in patterns of thinking, feeling, and behaving.
Big Five	A taxonomy for summarizing personality trait variation using five broad factors based on empirical patterns of covariation among people living in modern Western cultures; the Big Five personality traits are extraversion, agreeableness, conscientiousness, neuroticism, and openness to experience.
Genome	The complete set of genetic material in human cells, including nuclear DNA and a small amount of DNA in the mitochondria.
Single nucleotide polymorphism (SNP)	A location in the genome where a single nucleotide (A, C, T, or G) commonly varies across people.
**Genomic property**	
Genetic architecture	The structure of the patterning of genetic effects that underlie a given trait.
Locus	A location on the genome; typically, it refers to a region of physically close variants that are highly correlated. Independent loci are regions on the genome that are approximately statistically uncorrelated.
Linkage disequilibrium	Correlations between alleles at different genetic loci due to physical proximity on the genome or to population history.
Heritability	The proportion of trait variance that is attributable to genetic factors. When heritability is estimated using GWAS data, it is typically referred to as SNP heritability.
Polygenicity	The degree to which a trait’s heritability is associated with many genetic variants.
Pleiotropy	The extent to which genetic variants that are associated with one trait are also associated with other traits.
**Genomic method**	
Genetic linkage study	A method to identify trait-relevant genes by tracking how they are inherited together across generations or within sibling pairs.
Candidate gene study	A (now largely outdated) confirmatory method that tests associations between genetic variation at a specific locus and variation in a trait.
Genome-wide association study (GWAS)	An exploratory method that tests associations between millions of genetic variants across the genome and variation in a trait, with a strict significance threshold to reduce false positive findings.
Genome-wide significance threshold	In a GWAS, the *p*-value cutoff for concluding whether a genetic association with a trait is statistically significant; typically, it is *p* < 5 × 10^−8^ (0.00000005).
Polygenic index (PGI)	An individual value indexing genetic propensity for a trait, also called polygenic risk score (PRS) or polygenic score (PGS). First, a set of SNPs are selected from an independent discovery GWAS (e.g., genome-wide significant SNP, or all SNPs) and weighted according to their effect size estimates from the GWAS. Then, an individual’s predicted genetic propensity toward a trait is summarized into a single value based on the sum of those weighted SNPs.
Genetic correlation (*r*_g_)	The correlation between genetic propensities to two different traits; put differently, an index of the extent to which the genome-wide profile of genetic associations with one trait corresponds with the genome-wide profile of genetic associations with a second trait. Genetic correlations can be estimated even between traits measured in separate participant samples.

**Table 2:** Five key observations in the genetic architecture of personality traits

Observation	Description
Massive polygenicity	Very many genetic variants are associated with personality traits. These variants are not localized to a small region of DNA but are spread widely across the genome.
Widespread pleiotropy	Genetic variants associated with personality are associated with many other behaviorally relevant human characteristics.
High generality	Associations between genetic variants and personality traits are highly similar, but not identical, across groupings of people.
Minimal familial confounding	Associations between genetic variants and personality traits are minimally affected by parental similarity in personality, population stratification, and shared family environment.
Cross-method replicability	The science of personality genetics is cumulative and complementary. Inferences from GWAS methodologies replicate and expand inferences derived from twin and family methods.

## GENETIC VARIATION

The human genome is composed of paired combinations of four nucleotide bases: adenine (A), cytosine (C), guanine (G), and thymine (T), in a sequence approximately 3 billion bases long across 23 pairs of chromosomes (coils of DNA). Nearly the entire genome is the same across all humans: In only about 20 million locations do these nucleotides vary in ≥ 0.5% of people (The 1000 Genomes Project Consortium 2015). These commonly varying variants are called SNPs, pronounced “snips,” which is short for single nucleotide (i.e., a single DNA base pair) polymorphism (Greek for “many shapes,” referring to variation). For example, consider the 92,258,010th base pair on chromosome 6, a SNP numbered rs4360104 in Human Genome build 19. Variation is common at this spot in the genome, where the more common nucleotide is an A (paired to a T) and the less common nucleotide is a G (paired to a C) (Phan et al. 2020). Genomic variation has other properties as well: Each person’s genome also contains patterns of inserted and deleted segments of DNA, copy number variants, and rare genetic mutations, each of which may affect personality (e.g., Williams syndrome, characterized by an overfriendly, empathetic personality, is caused by a rare genetic deletion; Morris 1999). At present, genomic research on personality typically excludes these other forms of genetic variation.

## GENOME-WIDE ASSOCIATION STUDY BASICS

For the 22 pairs of autosomes, and the one pair of sex chromosomes, a person inherits one chromosome from each parent. Consider again the exemplar SNP rs4360104, where a person can inherit three possible combinations of alleles: an A from both mother and father, an A from one parent and a G from the other, or a G from both mother and father. SNPs are assessed in several ways. Some are measured directly using high-throughput microarray chips, and estimated values for others are imputed by integrating information from population reference genomes (e.g., International HapMap Consortium 2005, The 1000 Genomes Project Consortium 2015). In a GWAS, researchers conduct millions of association tests to identify whether variation in these measured and imputed SNPs predict individual differences in an attribute (Risch & Merikangas 1996). To minimize the number of false-positive results from these millions of tests, researchers impose stringent thresholds for statistical significance (typically *p* < 5 × 10^−8^, representing a 5% false discovery rate that has been corrected for approximately 1 million independent pieces of information in a standard GWAS; Pe’er et al. 2008).

## UNDERSTANDING GENOME-WIDE ASSOCIATION STUDY RESULTS

To maximize GWAS sample sizes, researchers form large consortia and combine data across many cohorts with meta-analysis. For example, in the most recent large-scale collaborative GWAS, over 140 collaborators in the Revived Genomics of Personality Consortium (Schwaba et al. 2025a) collected information on 11 million SNPs in up to 1.14 million participants with European-like and African-like genomes from 46 different study cohorts. They tested whether variation across these 11 million locations in the genome was associated with the Big Five personality traits and identified over 1,200 statistically distinct SNPs associated with one of the Big Five traits. For example, for SNP rs4360104, people who inherited more Gs (compared to As) tended to be significantly less extraverted (*b* = −0.017, SE = 0.002, *p* = 2.5 × 10^−20^, *R*^*2*^ = 0.00014; Schwaba et al. 2025a). Specifically, inheriting an additional G at this location in the genome was associated with a person scoring 0.017 standard deviations lower on extraversion, on average, after incorporating control variables, accounting for ~0.01% of the total measured variation in extraversion. This was one of the largest effect sizes detected, yet it is small. Generally, standardized associations around *r* = 0.10 are considered small in personality research (Funder & Ozer 2019),and this effect is roughly 1/10th the magnitude of that. In fact, it is likely an overestimate, as effects identified on the basis of their significance tend to be inflated (Forde et al. 2023). However, given the miniscule standard error and *p*-value, there is strong evidence that this association differs from zero.

## MANHATTAN PLOTS

In GWAS, genetic associations across the genome are depicted visually in a Manhattan plot, like the one inset in Figure 1, in which rs4360104 is highlighted. A Manhattan plot is so named because a well-powered set of GWAS results will resemble the Manhattan skyline, with many individual towers. Each tower is composed of many SNPs whose effects are intercorrelated due to a property of the genome called linkage disequilibrium: Genetic variants, typically those nearby in the genome, are inherited in chunks, not allele-by-allele. Thus, neighboring SNPs tend to have similar GWAS associations because they are highly collinear. To simplify the reporting of these genetic effects, researchers often tally the number of independently inherited loci that contain a genome-wide significant SNP (i.e., they count the genome-wide significant towers in a Manhattan plot rather than the individual genome-wide significant SNPs) and report a lead SNP with the lowest *p*-value in the locus. In aggregate, the small effects of SNPs throughout the genome account for substantial variance in personality traits. Schwaba et al. (2025a) estimate the SNP heritability of extraversion scores at approximately 9%. This means that about 9% of the variance in extraversion scores is attributable to genetic signal tagged by common SNPs.

## References

[R1] Abdellaoui A, Dolan CV, Verweij KJ, Nivard MG. 2022. Gene–environment correlations across geographic regions affect genome-wide association studies. Nat. Genet. 54(9):1345–54 35995948 10.1038/s41588-022-01158-0 PMC9470533

[R2] Abdellaoui A, Sanchez-Roige S, Sealock J, Treur JL, Dennis J, 2019. Phenome-wide investigation of health outcomes associated with genetic predisposition to loneliness. Hum. Mol. Genet. 28(22):3853–65 31518406 10.1093/hmg/ddz219 PMC6935385

[R3] Abdellaoui A, Yengo L, Verweij KJ, Visscher PM. 2023. 15 years of GWAS discovery: realizing the promise. Am. J. Hum. Genet. 110(2):179–94 36634672 10.1016/j.ajhg.2022.12.011 PMC9943775

[R4] Akingbuwa WA, Hammerschlag AR, Bartels M, Nivard MG, Middeldorp CM. 2022. Ultra-rare and common genetic variant analysis converge to implicate negative selection and neuronal processes in the aetiology of schizophrenia. Mol. Psychiatry 27(9):3699–707 35665764 10.1038/s41380-022-01621-8 PMC9708595

[R5] Allport GW, Odbert HS. 1936. Trait-names: a psycho-lexical study. Psychol. Monogr. 47(1):i–171

[R6] Atkinson EG, Maihofer AX, Kanai M, Martin AR, Karczewski KJ, 2021. Tractor uses local ancestry to enable the inclusion of admixed individuals in GWAS and to boost power. Nat. Genet. 53(2):195–204 33462486 10.1038/s41588-020-00766-y PMC7867648

[R7] Bainbridge TF, Ludeke SG, Smillie LD. 2022. Evaluating the Big Five as an organizing framework for commonly used psychological trait scales. J. Personal. Soc. Psychol. 122(4):749–77 10.1037/pspp0000395 35025595

[R8] Bakwin RM. 1930. Similarities and differences in identical twins. Pedag. Semin. J. Genet. Psychol. 38(1–4):373–97

[R9] Barlow DH, Sauer-Zavala S, Carl JR, Bullis JR, Ellard KK. 2014. The nature, diagnosis, and treatment of neuroticism: back to the future. Clin. Psychol. Sci. 2(3):344–65

[R10] Baselmans BM, Jansen R, Ip HF, van Dongen J, Abdellaoui A, 2019. Multivariate genome-wide analyses of the well-being spectrum. Nat. Genet. 51(3):445–51 30643256 10.1038/s41588-018-0320-8

[R11] Bernabeu E, Canela-Xandri O, Rawlik K, Talenti A, Prendergast J, Tenesa A. 2021. Sex differences in genetic architecture in the UK Biobank. Nat. Genet. 53(9):1283–89 34493869 10.1038/s41588-021-00912-0

[R12] Bogg T, Roberts BW. 2004. Conscientiousness and health-related behaviors: a meta-analysis of the leading behavioral contributors to mortality. Psychol. Bull. 130(6):887–919 15535742 10.1037/0033-2909.130.6.887

[R13] Boomsma DI, Helmer Q, Nieuwboer HA, Hottenga JJ, de Moor MH, 2018. An extended twin-pedigree study of neuroticism in the Netherlands Twin Register. Behav. Genet. 48:1–11 29043520 10.1007/s10519-017-9872-0 PMC5752751

[R14] Border R, Johnson EC, Evans LM, Smolen A, Berley N, 2019. No support for historical candidate gene or candidate gene-by-interaction hypotheses for major depression across multiple large samples. Am. J. Psychiatry 176(5):376–87 30845820 10.1176/appi.ajp.2018.18070881 PMC6548317

[R15] Borghans L, Golsteyn BH, Heckman JJ, Humphries JE. 2016. What grades and achievement tests measure. PNAS 113(47):13354–59 27830648 10.1073/pnas.1601135113 PMC5127298

[R16] Bouchard TJ Jr. 1994. Genes, environment, and personality. Science 264(5166):1700–1 8209250 10.1126/science.8209250

[R17] Bouchard TJ Jr., McGue M. 1990. Genetic and rearing environmental influences on adult personality: an analysis of adopted twins reared apart. J. Personal. 58(1):263–92 10.1111/j.1467-6494.1990.tb00916.x 23750380

[R18] Boyle EA, Li YI, Pritchard JK. 2017. An expanded view of complex traits: from polygenic to omnigenic. Cell 169(7):1177–86 28622505 10.1016/j.cell.2017.05.038 PMC5536862

[R19] Brainstorm Consortium, Anttila V, Bulik-Sullivan B, Finucane HK, Walters RK, 2018. Analysis of shared heritability in common disorders of the brain. Science 360(6395):eaap8757 29930110 10.1126/science.aap8757 PMC6097237

[R20] Bralten J, Mota NR, Klemann CJ, De Witte W, Laing E, 2021. Genetic underpinnings of sociability in the general population. Neuropsychopharmacology 46(9):1627–34 34054130 10.1038/s41386-021-01044-z PMC8280100

[R21] Briley DA, Livengood J, Derringer J. 2018. Behaviour genetic frameworks of causal reasoning for personality psychology. Eur. J. Personal. 32(3):202–20

[R22] Briley DA, Tucker-Drob EM. 2012. Broad bandwidth or high fidelity? Evidence from the structure of genetic and environmental effects on the facets of the five factor model. Behav. Genet. 42:743–63 22695681 10.1007/s10519-012-9548-8 PMC5380466

[R23] Briley DA, Tucker-Drob EM. 2014. Genetic and environmental continuity in personality development: a meta-analysis. Psychol. Bull. 140(5):1303–31 24956122 10.1037/a0037091 PMC4152379

[R24] Bryois J, Skene NG, Hansen TF, Kogelman LJ, Watson HJ, 2020. Genetic identification of cell types underlying brain complex traits yields insights into the etiology of Parkinson’s disease. Nat. Genet. 52(5):482–93 32341526 10.1038/s41588-020-0610-9 PMC7930801

[R25] Bulik-Sullivan BK, Loh PR, Finucane HK, Ripke S, Yang J, 2015. LD Score regression distinguishes confounding from polygenicity in genome-wide association studies. Nat. Genet. 47(3):291–95 25642630 10.1038/ng.3211 PMC4495769

[R26] Burt CH. 2024. Polygenic indices (aka polygenic scores) in social science: a guide for interpretation and evaluation. Sociol. Methodol. 54(2). 10.1177/0081175024123648 PMC11293310 39091537

[R27] Cardon LR, Palmer LJ. 2003. Population stratification and spurious allelic association. Lancet 361(9357):598–604 12598158 10.1016/S0140-6736(03)12520-2

[R28] Carroll JB. 1993. Human Cognitive Abilities: A Survey of Factor-Analytic Studies. Cambridge University Press

[R29] Caspi A, Sugden K, Moffitt TE, Taylor A, Craig IW, 2003. Influence of life stress on depression: moderation by a polymorphism in the 5-HTT gene. Science 301(5631):386–89 12869766 10.1126/science.1083968

[R30] Cheverud JM. 1984. Quantitative genetics and developmental constraints on evolution by selection. J. Theor. Biol. 110(2):155–71 6492829 10.1016/s0022-5193(84)80050-8

[R31] Clapp Sullivan ML, Schwaba T, Harden KP, Grotzinger AD, Nivard MG, Tucker-Drob EM. 2024. Beyond the factor indeterminacy problem using genome-wide association data. Nat. Hum. Behav. 8(2):205–18 38225407 10.1038/s41562-023-01789-1 PMC10922726

[R32] Cordioli M, Corbetta A, Kariis HM, Jukarainen S, Vartiainen P, 2024. Socio-demographic and genetic risk factors for drug adherence and persistence across 5 common medication classes. Nat. Commun. 15(1):9156 39443518 10.1038/s41467-024-53556-z PMC11500092

[R33] Costa PT, McCrae RR. 1992a. Neo Personality Inventory–Revised (NEO PI-R). Psychological Assessment Resources

[R34] Costa PT, McCrae RR. 1992b. Revised NEO Personality Inventory (NEO PI-R) and NEO Five-Factor Inventory (NEO FFI): Professional Manual. Psychological Assessment Resources

[R35] Costa PT Jr., McCrae RR, Löckenhoff CE. 2019. Personality across the life span. Annu. Rev. Psychol. 70:423–48 30231002 10.1146/annurev-psych-010418-103244

[R36] Craddock NJ, Jones IR, The Wellcome Trust Case Control Consortium. 2007. Genome-wide association study of 14,000 cases of seven common diseases and 3,000 shared controls. Nature 447:661–78 17554300 10.1038/nature05911 PMC2719288

[R37] Cramer AO, Van der Sluis S, Noordhof A, Wichers M, Geschwind N, 2012. Dimensions of normal personality as networks in search of equilibrium: You can’t like parties if you don’t like people. Eur. J. Personal. 26(4):414–31

[R38] Day FR, Ong KK, Perry JR. 2018. Elucidating the genetic basis of social interaction and isolation. Nat. Commun. 9(1):2457 29970889 10.1038/s41467-018-04930-1 PMC6030100

[R39] De Moor MH, Costa PT, Terracciano A, Krueger RF, De Geus EJ, 2012. Meta-analysis of genome-wide association studies for personality. Mol. Psychiatry 17(3):337–49 21173776 10.1038/mp.2010.128 PMC3785122

[R40] De Moor MH, Van Den Berg SM, Verweij KJ, Krueger RF, Luciano M, 2015. Meta-analysis of genome-wide association studies for neuroticism, and the polygenic association with major depressive disorder. JAMA Psychiatry 72(7):642–50 25993607 10.1001/jamapsychiatry.2015.0554 PMC4667957

[R41] Deelen J, Evans DS, Arking DE, Tesi N, Nygaard M, 2019. A meta-analysis of genome-wide association studies identifies multiple longevity genes. Nat. Commun. 10(1):3669 31413261 10.1038/s41467-019-11558-2 PMC6694136

[R42] DeYoung CG. 2015. Cybernetic big five theory. J. Res. Personal. 56:33–58

[R43] Eaves L, Eysenck H. 1975. The nature of extraversion: a genetical analysis. J. Personal. Soc. Psychol. 32(1):102–12 10.1037/h0076862 1239499

[R44] Ebert T, Gebauer JE, Brenner T, Bleidorn W, Gosling SD, 2022. Are regional differences in psychological characteristics and their correlates robust? Applying spatial-analysis techniques to examine regional variation in personality. Perspect. Psychol. Sci. 17(2):407–41 34699736 10.1177/1745691621998326

[R45] Eysenck HJ, Prell DB. 1951. The inheritance of neuroticism: an experimental study. J. Mental Sci. 97:441–65 10.1192/bjp.97.408.441 14861606

[R46] Fisher RA. 1918. The correlation between relatives on the supposition of Mendelian inheritance. Trans. R. Soc. Edinburgh 52(2):399–33

[R47] Forde A, Hemani G, Ferguson J. 2023. Review and further developments in statistical corrections for Winner’s Curse in genetic association studies. PLOS Genet. 19(9):e1010546 37721937 10.1371/journal.pgen.1010546 PMC10538662

[R48] Franić S, Dolan CV, Borsboom D, Hudziak JJ, van Beijsterveldt CE, Boomsma DI. 2013. Can genetics help psychometrics? Improving dimensionality assessment through genetic factor modeling. Psychol. Methods 18(3):406–33 23834420 10.1037/a0032755

[R49] Funder DC, Ozer DJ. 2019. Evaluating effect size in psychological research: sense and nonsense. Adv. Methods Pract. Psychol. Sci. 2(2):156–68

[R50] Gage SH, Smith GD, Ware JJ, Flint J, Munafo MR. 2016. G = E: what GWAS can tell us about the environment. PLOS Genet. 12(2):e1005765 26866486 10.1371/journal.pgen.1005765 PMC4750859

[R51] Gelman A 2018. You need 16 times the sample size to estimate an interaction than to estimate a main effect. Statistical Modeling, Causal Inference, and Social Science. https://statmodeling.stat.columbia.edu/2018/03/15/need16/

[R52] Goldberg LR. 1990. An alternative “description of personality”: the big-five factor structure. J. Personal. Soc. Psychol. 59(6):1216–29 10.1037//0022-3514.59.6.1216 2283588

[R53] Gottesman II. 1963. Heritability of personality: a demonstration. Psychol. Monogr. Gen. Appl. 77(9):1–21 10.1037/h0093852 4381911

[R54] Grosz MP, Rohrer JM, Thoemmes F. 2020. The taboo against explicit causal inference in nonexperimental psychology. Perspect. Psychol. Sci. 15(5):1243–55 32727292 10.1177/1745691620921521 PMC7472833

[R55] Grotzinger AD, Rhemtulla M, de Vlaming R, Ritchie SJ, Mallard TT, 2019. Genomic structural equation modelling provides insights into the multivariate genetic architecture of complex traits. Nat. Hum. Behav. 3(5):513–25 30962613 10.1038/s41562-019-0566-x PMC6520146

[R56] Grotzinger AD, Werme J, Peyrot WJ, Frei O, de Leeuw C, 2025. The landscape of shared and divergent genetic influences across 14 psychiatric disorders. Preprint, medRxiv 2025.01.14.25320574

[R57] Gupta P, Galimberti M, Liu Y, Beck S, Wingo A, 2024. A genome-wide investigation into the underlying genetic architecture of personality traits and overlap with psychopathology. Nat. Hum. Behav. 8:2235–49 39134740 10.1038/s41562-024-01951-3 PMC11576509

[R58] Hariri AR, Mattay VS, Tessitore A, Kolachana B, Fera F, 2002. Serotonin transporter genetic variation and the response of the human amygdala. Science 297(5580):400–3 12130784 10.1126/science.1071829

[R59] Hill WD, Weiss A, Liewald DC, Davies G, Porteous DJ, 2020. Genetic contributions to two special factors of neuroticism are associated with affluence, higher intelligence, better health, and longer life. Mol. Psychiatry 25(11):3034–52 30867560 10.1038/s41380-019-0387-3 PMC7577854

[R60] Horwitz TB, Balbona JV, Paulich KN, Keller MC. 2023. Evidence of correlations between human partners based on systematic reviews and meta-analyses of 22 traits and UK Biobank analysis of 133 traits. Nat. Hum. Behav. 7(9):1568–83 37653148 10.1038/s41562-023-01672-z PMC10967253

[R61] Howe LJ, Nivard MG, Morris TT, Hansen AF, Rasheed H, 2022. Within-sibship genome-wide association analyses decrease bias in estimates of direct genetic effects. Nat. Genet. 54(5):581–92 35534559 10.1038/s41588-022-01062-7 PMC9110300

[R62] Hutchinson A, Asimit J, Wallace C. 2020. Fine-mapping genetic associations. Hum. Mol. Genet. 29(R1):R81–88 32744321 10.1093/hmg/ddaa148 PMC7733401

[R63] Hyatt CS, Sharpe BM, Owens MM, Listyg BS, Carter NT, 2022. Searching high and low for meaningful and replicable morphometric correlates of personality. J. Personal. Soc. Psychol. 123(2):463–80 10.1037/pspp0000402 34766808

[R64] International HapMap Consortium. 2005. A haplotype map of the human genome. Nature 437:1299–320 16255080 10.1038/nature04226 PMC1880871

[R65] Jang KL, McCrae RR, Angleitner A, Riemann R, Livesley WJ. 1998. Heritability of facet-level traits in a cross-cultural twin sample: support for a hierarchical model of personality. J. Personal. Soc. Psychol. 74(6):1556–65 10.1037//0022-3514.74.6.1556 9654759

[R66] John OP, Naumann LP, Soto CJ. 2008. Paradigm shift to the integrative Big Five trait taxonomy: history, measurement, and conceptual issues. In Handbook of Personality: Theory and Research, ed. John OP, Robins RW, Pervin LA, pp. 114–58. Guilford Press. 3rd ed.

[R67] Jokela M, Bleidorn W, Lamb ME, Gosling SD, Rentfrow PJ. 2015. Geographically varying associations between personality and life satisfaction in the London metropolitan area. PNAS 112(3):725–30 25583480 10.1073/pnas.1415800112 PMC4311843

[R68] Kandler C, Bratko D, Butković A, Hlupić TV, Tybur JM, 2021. How genetic and environmental variance in personality traits shift across the life span: evidence from a cross-national twin study. J. Personal. Soc. Psychol. 121(5):1079–94 10.1037/pspp0000366 32969676

[R69] Kandler C, Riemann R, Spinath FM, Angleitner A. 2010. Sources of variance in personality facets: a multiple-rater twin study of self-peer, peer-peer, and self-self (dis) agreement. J. Personal. 78(5):1565–94 10.1111/j.1467-6494.2010.00661.x 20663023

[R70] Karlsson Linnér R, Biroli P, Kong E, Meddens SFW, Wedow R, 2019. Genome-wide association analyses of risk tolerance and risky behaviors in over 1 million individuals identify hundreds of loci and shared genetic influences. Nat. Genet. 51(2):245–57 30643258 10.1038/s41588-018-0309-3 PMC6713272

[R71] Karlsson Linnér R, Mallard TT, Barr PB, Sanchez-Roige S, Madole JW, 2021. Multivariate analysis of 1.5 million people identifies genetic associations with traits related to self-regulation and addiction. Nat. Neurosci. 24(10):1367–76 34446935 10.1038/s41593-021-00908-3 PMC8484054

[R72] Kendler KS, Lönn SL, Maes HH, Lichtenstein P, Sundquist J, Sundquist K. 2015. A Swedish population-based multivariate twin study of externalizing disorders. Behav. Genet. 46:183–92 26494460 10.1007/s10519-015-9741-7 PMC4752872

[R73] Kim BH, Kim HN, Roh SJ, Lee MK, Yang S, 2015. GWA meta-analysis of personality in Korean cohorts. J. Hum. Genet. 60(8):455–60 25994864 10.1038/jhg.2015.52

[R74] Kim Y, Saunders GR, Giannelis A, Willoughby EA, DeYoung CG, Lee JJ. 2023. Genetic and neural bases of the neuroticism general factor. Biol. Psychol. 184:108692 37783279 10.1016/j.biopsycho.2023.108692

[R75] Kyaga S, Lichtenstein P, Boman M, Hultman C, Långström N, Landen M. 2011. Creativity and mental disorder: family study of 300 000 people with severe mental disorder. Br. J. Psychiatry 199(5):373–79 21653945 10.1192/bjp.bp.110.085316

[R76] Lee K, Ashton MC. 2004. Psychometric properties of the HEXACO personality inventory. Multivar. Behav. Res. 39(2):329–58 10.1207/s15327906mbr3902_8 26804579

[R77] Lesch KP, Bengel D, Heils A, Sabol SZ, Greenberg BD, 1996. Association of anxiety-related traits with a polymorphism in the serotonin transporter gene regulatory region. Science 274(5292):1527–31 8929413 10.1126/science.274.5292.1527

[R78] Lo MT, Hinds DA, Tung JY, Franz C, Fan CC, 2017. Genome-wide analyses for personality traits identify six genomic loci and show correlations with psychiatric disorders. Nat. Genet. 49(1):152–56 27918536 10.1038/ng.3736 PMC5278898

[R79] Loehlin JC. 1992. Genes and Environment in Personality Development. Sage

[R80] Luciano M, Hagenaars SP, Davies G, Hill WD, Clarke TK, 2018. Association analysis in over 329,000 individuals identifies 116 independent variants influencing neuroticism. Nat. Genet. 50(1):6–11 29255261 10.1038/s41588-017-0013-8 PMC5985926

[R81] Luhmann M, Fassbender I, Alcock M, Haehner P. 2021. A dimensional taxonomy of perceived characteristics of major life events. J. Personal. Soc. Psychol. 121(3):633–68 10.1037/pspp0000291 32338939

[R82] Lundberg M, Sng LM, Szul P, Dunne R, Bayat A, 2023. Novel Alzheimer’s disease genes and epistasis identified using machine learning GWAS platform. Sci. Rep. 13(1):17662 37848535 10.1038/s41598-023-44378-y PMC10582044

[R83] Madole JW, Harden KP. 2023. Building causal knowledge in behavior genetics. Behav. Brain Sci. 46:e182 10.1017/S0140525X22000681 35510303

[R157] Maher B. 2008. The case of the missing heritability. Nature 456(7218):18–21 18987709 10.1038/456018a

[R84] Mann FD, Patterson MW, Grotzinger AD, Kretsch N, Tackett JL, 2016. Sensation seeking, peer deviance, and genetic influences on adolescent delinquency: evidence for person-environment correlation and interaction. J. Abnorm. Psychol. 125(5):679–91 27124714 10.1037/abn0000160 PMC8256371

[R85] Matthews LJ, Turkheimer E. 2022. Three legs of the missing heritability problem. Stud. Hist. Philos. Sci. 93:183–91 35533541 10.1016/j.shpsa.2022.04.004 PMC9172633

[R86] Mbatchou J, Barnard L, Backman J, Marcketta A, Kosmicki JA, 2021. Computationally efficient whole-genome regression for quantitative and binary traits. Nat. Genet. 53(7):1097–103 34017140 10.1038/s41588-021-00870-7

[R87] McAdams DP, Olson BD. 2010. Personality development: continuity and change over the life course. Annu. Rev. Psychol. 61:517–42 19534589 10.1146/annurev.psych.093008.100507

[R88] McCrae RR, Martin TA, Hrebícková M, Urbánek T, Boomsma DI, Willemsen G, Costa PT Jr. 2008. Personality trait similarity between spouses in four cultures. J. Personal. 76(5):1137–64 10.1111/j.1467-6494.2008.00517.x PMC2626346 18665894

[R89] Molenaar PC, Boomsma DI, Dolan CV. 1993. A third source of developmental differences. Behav. Genet. 23:519–24 8129693 10.1007/BF01068142

[R90] Morris CA. 1999. Williams syndrome. In GeneReviews, ed. Adam MP, Feldman J, Mirzaa GM, Pagon RA, Wallace SE, Amemiya A. University of Washington, Seattle 20301427

[R91] Mõttus R, Kandler C, Bleidorn W, Riemann R, McCrae RR. 2017. Personality traits below facets: the consensual validity, longitudinal stability, heritability, and utility of personality nuances. J. Personal. Soc. Psychol. 112(3):474–90 10.1037/pspp0000100 27124378

[R92] Mõttus R, Kandler C, Luciano M, Esko T, Vainik U. 2025. Familial transmission of personality traits and life satisfaction is much higher than shown in typical single-method studies. J. Personal. Soc. Psychol. 128(6):1336–54 10.1037/pspp0000550 40146588

[R93] Nagel M, Jansen PR, Stringer S, Watanabe K, De Leeuw CA, 2018. Meta-analysis of genome-wide association studies for neuroticism in 449,484 individuals identifies novel genetic loci and pathways. Nat. Genet. 50(7):920–27 29942085 10.1038/s41588-018-0151-7

[R94] Neale MCCL, Cardon LR. 2013. Methodology for Genetic Studies of Twins and Families. Springer

[R95] Noftle EE, Robins RW. 2007. Personality predictors of academic outcomes: Big Five correlates of GPA and SAT scores. J. Personal. Soc. Psychol. 93(1):116–30 10.1037/0022-3514.93.1.116 17605593

[R96] Novembre J, Stephens M. 2008. Interpreting principal component analyses of spatial population genetic variation. Nat. Genet. 40(5):646–49 18425127 10.1038/ng.139 PMC3989108

[R97] Okbay A, Baselmans BM, De Neve JE, Turley P, Nivard MG, 2016. Genetic variants associated with subjective well-being, depressive symptoms, and neuroticism identified through genome-wide analyses. Nat. Genet. 48(6):624–33 27089181 10.1038/ng.3552 PMC4884152

[R98] Okbay A, Wu Y, Wang N, Jayashankar H, Bennett M, 2022. Polygenic prediction of educational attainment within and between families from genome-wide association analyses in 3 million individuals. Nat. Genet. 54(4):437–49 35361970 10.1038/s41588-022-01016-z PMC9005349

[R99] Palmer DS, Zhou W, Abbott L, Wigdor EM, Baya N, 2023. Analysis of genetic dominance in the UK Biobank. Science 379(6639):1341–48 36996212 10.1126/science.abn8455 PMC10345642

[R100] Pasaniuc B, Price AL. 2017. Dissecting the genetics of complex traits using summary association statistics. Nat. Rev. Genet. 18(2):117–27 27840428 10.1038/nrg.2016.142 PMC5449190

[R101] Pasman JA, Chen Z, Smit DJ, Vink JM, Van Den Oever MC, 2022. The CADM2 gene and behavior: a phenome-wide scan in UK-Biobank. Behav. Genet. 52(4):306–14 35867259 10.1007/s10519-022-10109-8 PMC9463269

[R102] Paunonen SV, Jackson DN. 2000. What is beyond the Big Five? Plenty! J. Personal. 68(5):821–35 10.1111/1467-6494.00117 11001150

[R103] Pearson K 1904. On the laws of inheritance in man: II. On the inheritance of the mental and moral characters in man, and its comparison with the inheritance of the physical characters. Biometrika 3(2/3):131–90

[R104] Pe’er I, Yelensky R, Altshuler D, Daly MJ. 2008. Estimation of the multiple testing burden for genomewide association studies of nearly all common variants. Genet. Epidemiol. 32(4):381–85 18348202 10.1002/gepi.20303

[R105] Penke L, Jokela M. 2016. The evolutionary genetics of personality revisited. Curr. Opin. Psychol. 7:104–9

[R106] Phan L, Jin Y, Zhang H, Qiang W, Shekhtman D, 2020, Mar. 10. ALFA: Allele Frequency Aggregator. National Library of Medicine. https://www.ncbi.nlm.nih.gov/snp/docs/gsr/alfa/

[R107] Plomin R, Daniels D. 1987. Why are children in the same family so different from one another? Behav. Brain Sci. 10(1):1–16

[R108] Plomin R, DeFries JC, Loehlin JC. 1977. Genotype-environment interaction and correlation in the analysis of human behavior. Psychol. Bull. 84(2):309–22 557211

[R109] Polderman TJ, Benyamin B, De Leeuw CA, Sullivan PF, Van Bochoven A, 2015. Meta-analysis of the heritability of human traits based on fifty years of twin studies. Nat. Genet. 47(7):702–9 25985137 10.1038/ng.3285

[R110] Power GM, Sanderson E, Pagoni P, Fraser A, Morris T, 2024. Methodological approaches, challenges, and opportunities in the application of Mendelian randomisation to lifecourse epidemiology: a systematic literature review. Eur. J. Epidemiol. 39(5):501–20 37938447 10.1007/s10654-023-01032-1 PMC7616129

[R111] Power RA, Steinberg S, Bjornsdottir G, Rietveld CA, Abdellaoui A, 2015. Polygenic risk scores for schizophrenia and bipolar disorder predict creativity. Nat. Neurosci. 18(7):953–55 26053403 10.1038/nn.4040

[R112] Purcell S 2002. Variance components models for gene–environment interaction in twin analysis. Twin Res. Hum. Genet. 5(6):554–71 10.1375/136905202762342026 12573187

[R113] Risch N, Merikangas K. 1996. The future of genetic studies of complex human diseases. Science 273(5281):1516–17 8801636 10.1126/science.273.5281.1516

[R114] Roberts BW, Kuncel NR, Shiner R, Caspi A, Goldberg LR. 2007. The power of personality: the comparative validity of personality traits, socioeconomic status, and cognitive ability for predicting important life outcomes. Perspect. Psychol. Sci. 2(4):313–45 26151971 10.1111/j.1745-6916.2007.00047.x PMC4499872

[R115] Roberts BW, Yoon HJ. 2022. Personality psychology. Annu. Rev. Psychol. 73:489–516 34516758 10.1146/annurev-psych-020821-114927

[R116] Rukh G, de Ruijter M, Schiöth HB. 2023. Effect of worry, depression, and sensitivity to environmental stress owing to neurotic personality on risk of cardiovascular disease: a Mendelian randomization study. J. Personal. 91(3):856–67 10.1111/jopy.12782 36165189

[R117] Sanchez-Roige S, Jennings MV, Thorpe HH, Mallari JE, van der Werf LC, 2023. CADM2 is implicated in impulsive personality and numerous other traits by genome-and phenome-wide association studies in humans and mice. Transl. Psychiatry 13(1):167 37173343 10.1038/s41398-023-02453-y PMC10182097

[R118] Sanderson E, Glymour MM, Holmes MV, Kang H, Morrison J, 2022. Mendelian randomization. Nat. Rev. Methods Primers 2(1):6 37325194 10.1038/s43586-021-00092-5 PMC7614635

[R119] Schwaba T, Clapp Sullivan ML, Akingbuwa WA, Tanksley PT, Williams CM, 2025a. Robust inference and widespread genetic correlates from a large-scale genetic association study of human personality. Preprint, bioRxiv 2025.05.16.648988

[R120] Schwaba T, Mallard TT, Maihofer AX, Rhemtulla M, Lee PH, 2025b. Comparison of the multivariate genetic architecture of eight major psychiatric disorders across sex. Nat. Genet. 57:583–90 40055480 10.1038/s41588-025-02093-6 PMC12022846

[R121] Smith GD. 2011. Epidemiology, epigenetics and the “Gloomy Prospect”: embracing randomness in population health research and practice. Int. J. Epidemiol. 40(3):537–62 21807641 10.1093/ije/dyr117

[R122] Smith SP, Darnell G, Udwin D, Stamp J, Harpak A, 2024. Discovering non-additive heritability using additive GWAS summary statistics. eLife 13:e90459 38913556 10.7554/eLife.90459 PMC11196113

[R123] Sniekers S, Stringer S, Watanabe K, Jansen PR, Coleman JR, 2017. Genome-wide association meta-analysis of 78,308 individuals identifies new loci and genes influencing human intelligence. Nat. Genet. 49(7):1107–12 28530673 10.1038/ng.3869 PMC5665562

[R124] Solovieff N, Cotsapas C, Lee PH, Purcell SM, Smoller JW. 2013. Pleiotropy in complex traits: challenges and strategies. Nat. Rev. Genet. 14(7):483–95 23752797 10.1038/nrg3461 PMC4104202

[R125] Soto CJ. 2019. How replicable are links between personality traits and consequential life outcomes? The life outcomes of personality replication project. Psychol. Sci. 30(5):711–27 30950321 10.1177/0956797619831612

[R126] Soto CJ, John OP. 2017. The next Big Five Inventory (BFI-2): developing and assessing a hierarchical model with 15 facets to enhance bandwidth, fidelity, and predictive power. J. Personal. Soc. Psychol. 113(1):117–43 10.1037/pspp0000096 27055049

[R127] Spearman C 1904. The proof and measurement of association between two things. Am. J. Psychol. 15(1):72–101 3322052

[R128] Srivastava S 2020. Personality structure: Who cares? Eur. J. Personal. 34(4):550–51

[R129] Streit F, Awasthi S, Hall AS, Niarchou M, Marouli E, 2024. Genome-wide association study of borderline personality disorder identifies six loci and highlights shared risk with mental and somatic disorders. Preprint, medRxiv 2024.11.12.24316957

[R130] Sun J, Vazire S. 2019. Do people know what they’re like in the moment? Psychol. Sci. 30(3):405–14 30653411 10.1177/0956797618818476

[R131] Sunde HF, Eftedal NH, Cheesman R, Corfield EC, Kleppesto TH, 2024. Genetic similarity between relatives provides evidence on the presence and history of assortative mating. Nat. Commun. 15(1):2641 38531929 10.1038/s41467-024-46939-9 PMC10966108

[R132] Sundin ZW, Chopik WJ, Welker KM, Ascigil E, Brandes CM, 2021. Estimating the associations between Big Five personality traits, testosterone, and cortisol. Adapt. Hum. Behav. Physiol. 7(3):307–40

[R133] Tan T, Jayashankar H, Guan J, Nehzati SM, Mir M, 2024. Family-GWAS reveals effects of environment and mating on genetic associations. Preprint, medRxiv 2024.10.01.24314703

[R134] Tanksley PT, Brislin SJ, Wertz J, de Vlaming R, Courchesne-Krak NS, 2025. Do polygenic indices capture “direct” effects on child externalizing behavior problems? Within-family analyses in two longitudinal birth cohorts. Clin. Psychol. Sci. 13(2):316–31 40110515 10.1177/21677026241260260 PMC11922333

[R135] Tellegen A, Lykken DT, Bouchard TJ, Wilcox KJ, Segal NL, Rich S. 1988. Personality similarity in twins reared apart and together. J. Personal. Soc. Psychol. 54(6):1031–9 10.1037//0022-3514.54.6.1031 3397862

[R136] Terracciano A, Sanna S, Uda M, Deiana B, Usala G, 2010. Genome-wide association scan for five major dimensions of personality. Mol. Psychiatry 15(6):647–56 18957941 10.1038/mp.2008.113 PMC2874623

[R137] Thalmayer AG, Saucier G, Rotzinger JS. 2022. Absolutism, relativism, and universalism in personality traits across cultures: the case of the Big Five. J. Cross-Cult. Psychol. 53(7–8):935–56

[R138] The 1000 Genomes Project Consortium. 2015. A global reference for human genetic variation. Nature 526:68–74 26432245 10.1038/nature15393 PMC4750478

[R139] Tropf FC, Lee SH, Verweij RM, Stulp G, Van Der Most PJ, De Vlaming R, 2017. Hidden heritability due to heterogeneity across seven populations. Nat. Hum. Behav. 1(10):757–65 29051922 10.1038/s41562-017-0195-1 PMC5642946

[R140] Turkheimer E, Pettersson E, Horn EE. 2014. A phenotypic null hypothesis for the genetics of personality. Annu. Rev. Psychol. 65:515–40 24050184 10.1146/annurev-psych-113011-143752

[R141] van de Weijer MP, Pelt DH, de Vries LP, Baselmans BM, Bartels M. 2022. A re-evaluation of candidate gene studies for well-being in light of genome-wide evidence. J. Happiness Stud. 23(6):3031–53 35949913 10.1007/s10902-022-00538-x PMC9356956

[R142] van den Berg SM, de Moor MH, Verweij KJ, Krueger RF, Luciano M, 2016. Meta-analysis of genome-wide association studies for extraversion: findings from the genetics of personality consortium. Behav. Genet. 46:170–82 26362575 10.1007/s10519-015-9735-5 PMC4751159

[R143] VanderWeele TJ, Vansteelandt S. 2022. A statistical test to reject the structural interpretation of a latent factor model. J. R. Stat. Soc. B 84(5):2032–54 10.1111/rssb.12555 PMC9937555 36818188

[R144] Veller C, Coop GM. 2024. Interpreting population-and family-based genome-wide association studies in the presence of confounding. PLOS Biol. 22(4):e3002511 38603516 10.1371/journal.pbio.3002511 PMC11008796

[R145] Verweij N, van de Vegte YJ, van der Harst P. 2018. Genetic study links components of the autonomous nervous system to heart-rate profile during exercise. Nat. Commun. 9(1):898 29497042 10.1038/s41467-018-03395-6 PMC5832790

[R146] Vukasović T, Bratko D. 2015. Heritability of personality: a meta-analysis of behavior genetic studies. Psychol. Bull. 141(4):769–85 25961374 10.1037/bul0000017

[R147] Wainschtein P, Jain D, Zheng Z, Cupples LA, Shadyab AH, 2022. Assessing the contribution of rare variants to complex trait heritability from whole-genome sequence data. Nat. Genet. 54(3):263–73 35256806 10.1038/s41588-021-00997-7 PMC9119698

[R148] Weiner DJ, Nadig A, Jagadeesh KA, Dey KK, Neale BM, 2023. Polygenic architecture of rare coding variation across 394,783 exomes. Nature 614(7948):492–99 36755099 10.1038/s41586-022-05684-z PMC10614218

[R149] Widiger TA, Crego C. 2019. *HiTOP* thought disorder, *DSM-5* psychoticism, and five factor model openness. J. Res. Personal. 80:72–77

[R150] Willroth EC, Luo J, Atherton OE, Weston SJ, Drewelies J, 2023. Personality traits and healthcare use: a coordinated analysis of 15 international samples. J. Personal. Soc. Psychol. 125(3):629–48 10.1037/pspp0000465 PMC10524692 37338439

[R151] Wright AJ, Jackson JJ. 2023. Do changes in personality predict life outcomes? J. Personal. Soc. Psychol. 125(6):1495–518 10.1037/pspp0000472 37384463

[R152] Wu XR, Li ZY, Yang L, Liu Y, Fei CJ, 2025. Large-scale exome sequencing identified 18 novel genes for neuroticism in 394,005 UK-based individuals. Nat. Hum. Behav. 9:406–19 39511343 10.1038/s41562-024-02045-w

[R153] Yang J, Bakshi A, Zhu Z, Hemani G, Vinkhuyzen AA, 2015. Genetic variance estimation with imputed variants finds negligible missing heritability for human height and body mass index. Nat. Genet. 47(10):1114–20 26323059 10.1038/ng.3390 PMC4589513

[R154] Young AI. 2022. Discovering missing heritability in whole-genome sequencing data. Nat. Genet. 54(3):224–26 35273401 10.1038/s41588-022-01012-3

[R155] Young AI, Nehzati SM, Benonisdottir S, Okbay A, Jayashankar H, 2022. Mendelian imputation of parental genotypes improves estimates of direct genetic effects. Nat. Genet. 54(6):897–905 35681053 10.1038/s41588-022-01085-0 PMC9197765

[R156] Zhang Y, Qi G, Park JH, Chatterjee N. 2018. Estimation of complex effect-size distributions using summary-level statistics from genome-wide association studies across 32 complex traits. Nat. Genet. 50(9):1318–26 30104760 10.1038/s41588-018-0193-x

